# Combined analysis of differentially expressed lncRNAs and miRNAs in liver tissues of high-fat fed rabbits by transcriptome sequencing

**DOI:** 10.3389/fgene.2022.1000574

**Published:** 2022-10-07

**Authors:** Jie Wang, Meigui Wang, Jiahao Shao, Zheliang Liu, Chong Fu, Guanhe Chen, Kaisen Zhao, Hong Li, Wenqiang Sun, Xianbo Jia, Shiyi Chen, Songjia Lai

**Affiliations:** ^1^ College of Animal Science and Technology, Sichuan Agricultural University, Chengdu, Sichuan, China; ^2^ Farm Animal Genetic Resources Exploration and Innovation Key Laboratory of Sichuan Province, Sichuan Agricultural University, Chengdu, Sichuan, China

**Keywords:** rabbit, miRNA, lncRNA, HFD, molecular mechanism

## Abstract

High-fat diet could lead to a series of metabolic diseases, including obesity, and its mechanism is not clear. In this study, the rabbit individuals were fed with high-fat diet, the liver tissues were collected, high-throughput sequencing technology was used to reveal the expression of lncRNA and miRNA difference, and the molecular regulation mechanism of lncRNA-miRNA. A total of 24,615 DE lncRNAs and 52 DE miRNAs were identified, including 15 novel discovered DE miRNAs (5 upregulated and 10 downregulated). Furthermore, five miRNAs and three mRNAs were verified by qRT-PCR, and the results showed that the expression of the DE miRNAs and DE lncRNAs in the two groups was consistent with our sequencing results. GO and KEGG analyzed 7,57,139 target genes respectively, enriching the pathways related to lipid metabolism, including mucin O-glycan biosynthesis pathway, insulin resistance and glucagon signaling pathway. Moreover, 65 targeting relationships were obtained. Among them, LOC103348122/miR-450a-5p, LOC103350359/miR-450a-3p and LOC103350429/miR-148a-5p were proposed the first time. Significantly, LOC103348122/miR-450a-5p and LOC103350429/miR-148a-5p were related to lipid metabolism in the liver. This study is of great significance to the CeRNA regulatory network related to lipid metabolism in the liver of rabbits, and provides a basis for understanding hepatic steatosis in rabbits.

## Introduction

The World Health Organization (WHO) defined obesity as the body mass index (BMI) higher than 30 kg/m^2^. During the past two decades, obesity is spreading quickly not only in developed but also in developing countries (Wang *and* Wang*.*, 2018). Overall, obesity is a complex pathological process, and available and cheaper highly palatable and fat-dense foods are important contributors (Boyd and Gary, 2011; Llewellyn & Wardle, 2015). Obesity poses a series of medical diseases, such as fatty liver, cardiovascular disease, type 2 diabetes, etc, becoming a considerable public problem leading to serious effects on health (Sheng et al., 2019). Among these, fatty liver is a serious threat to people’s health, becoming the second largest liver disease after viral hepatitis. For healthy individuals, liver tissue contains a small amount of fat, such as triglyceride, phospholipid, and cholesterol, its weight is about 3%–5% of the liver weight. The liver is the central organ that responds to fat metabolism, and the fat in the liver mainly comes from food and peripheral adipose tissue (Lopes et al., 2020). When the fat content was more than 5% of the liver weight or morphological observation revealed obvious steatosis, the fatty liver was identified at diagnosis. Nowadays, the incidence of fatty liver is increasing, and the onset age is getting younger and younger (Iwamura, 1989; Sartini et al., 2015). Multiple works indicated that the proportion of fat accumulation inside the liver is associated with the development degree of obesity, but the mechanisms underlying fatty liver remain incomplete, which prevents the development of effective therapies beyond the control of nutrition and physical exercise (Sturm et al., 2012; Gao et al., 2013).

Non-coding RNA is represented by circRNA, miRNA, lncRNA, etc. These non-coding RNA and the interactions between them are important for the regulation of multiple life activities. For example, miRNA is a class of endogenous about 22 nt RNA molecules that play a gene-regulatory role by binding to the mRNA 3′-UTR of the coding gene to direct their function at the post-transcriptional level (Bartel, 2009). Zhang et al. (2013) found that increased miR-15b abundance in non-alcoholic fatty liver disease (NAFLD) models may lead to decreased cell proliferation and glucose consumption while inducing the storage of intracellular triglycerides, which are all hazards of HFD-induced fatty liver. Serum levels of miR-34a and miR-122 were found to be significantly higher among fatty liver patients and were positively correlated with VLDL-C and triglyceride levels (Salvoza et al., 2016). In addition, the function of lncRNA also has been identified, including functioning as miRNAs sponges, trans-acting through base pairing with target RNA, and trans-acting through protein binding to sequences motifs or RNA structures (Hui et al., 2019). Chen et al. (2018) found that knockdown of AK012226 by siRNA significantly reduced the lipid accumulation in the NCTC1469 cells treated with free fatty acids. Moreover, NEAT1 aggravated FFA-induced lipid accumulation in hepatocytes by regulating the c-Jun/SREBP1c axis by sponging miR-139-5p (SiSi et al., 2021). Overall, although the past decade output many scientific research for fatty liver research, the molecular mechanisms of HFD induced fatty liver by mediating the lncRNA-miRNA regulation axis require further research. Thus, we aim to investigate the profile of miRNAs and lncRNAs in HFD induced steatosis by sequencing and analyzing the liver tissue from the rabbits fed a CON or HFD to obtain new insights into lncRNA-miRNA molecular regulatory mechanism and contribute to the understanding of epigenetic mechanisms influencing fat metabolism in obese rabbit liver.

## Materials and methods

### Ethics statement

All experiments in the current work involving animals were performed under the direction of the Institutional Animal Care and Use Committee from the College of Animal Science and Technology, Sichuan Agricultural University, China (DKY-B2019302083).

### Animals

Thirty six female Tianfu black rabbits were raised in the rabbit farm of Sichuan Agricultural University and randomly divided into two groups. Eighteen rabbits were fed standard diet (CON), 18 rabbits were fed high-fat diet (HFD: 10% lard was added to CON), and the feed was supplied three times a day and free drinking water was used. Feed the individual in a clean iron cage (600 mm × 600 mm × 500 mm). After 5 weeks, three obese and the normal rabbits were randomly selected from each group and slaughtered under the conditions of animal welfare. The liver tissues were collected according to Yuan et al. (2018) method, stored in liquid nitrogen, and sent to Nuohe (https://magic.novogene.com/customer/main#/login) for sequencing.

### Hematoxylin-eosin staining

The morphological differences of the liver in CON and HFD groups were observed by Hematoxylin-eosin (HE) staining (Cardiff et al., 2014). Four gram of liver tissue was separated, cleaned with PBS, and then mixed with 10% neutral formalin fixative. The sample was fixed in 4% paraformaldehyde for 24 h and then washed with sterile water. Secondly, the specimens were successively de-hydrated and embedded in paraffin. Then, a microtome (RM2235, Leica, Nussloch, Germany) was used to get the 5-µm-thick sections. Finally, images were captured using an inverted microscope (Olympus, Tokyo, Japan).

### RNA extraction and quantitative real-time PCR

Total RNA from the liver sample was extracted using RNAiso Plus Reagent (Invitrogen, Hong Kong, China), following the guidelines of the manufacturer. Subsequently, the purity, concentration, and integrity of RNA were determined by Agilent 2100 Bio-analyzer system (Agilent Technologies, Carlsbad, CA, United States), and only RNA meeting quality criteria (A260/A280 = 1.6–1.8; concentration ≥200 ng/μl) was used for the trial. Reverse transcription of mRNA and miRNA was performed using the RT EasyTM II (With gDNase) (FOREGENE, Chengdu, China) and the Mir-XTM miRNA First-Strand Synthesis Kit (Takara, Dalian, China), respectively. Then, qRT-PCR was performed in triplicate using the 2*TSINGKE Master qPCR Mix (SYBR Green I) (Tsingke, Chengdu, China) on a CFX96 instrument (Bio-Rad, Hercules, CA, United States), and the relative levels of mRNA and miRNA were calculated using the 2^−ΔΔCt^ method. The mRQ 3′ primer in the Mir-XTM miRNA First-Strand Synthesis Kit (Takara, Dalian, China) was served as a reverse primer for miRNA quantification, and U6 was used as an internal reference. Besides, GAPHD was used as the internal reference for mRNA quantification. The sequences of primers were showed in Table 1 and synthesized by Gene Pharma (Shanghai, China).

**TABLE 1:** Primer information for each gene.

Gene symbol		Primer sequences (5′ → 3′)
LOC103350359	Sense	GGC​CCC​TAG​CAT​GCA​GTT​TT
LOC103350359	Antisense	GGT​CCC​ATG​AGT​GTC​TCT​GC
LOC103348122	Sense	CTA​CTC​GCC​ACC​CAC​ACT​TT
LOC103348122	Antisense	CTC​CCA​ACA​GGT​GAG​CCA​AT
LOC103350429	Sense	GAT​CGA​GCC​ATT​GCG​TTT​CC
LOC103350429	Antisense	AAG​CCT​TTT​CTC​CTC​CTC​GC
LOC108178671	Sense	CTA​TGC​CAG​CGT​GAG​AAC​CAA
LOC108178671	Antisense	GCG​ATG​CTT​AGT​AAA​CGG​GTG
LOC108176670	Sense	ATC​GTC​CTC​TCC​CTA​ACA​TCA​CC
LOC108176670	Antisense	AAC​CTC​AGT​CCT​CCT​GCC​GC
LOC108178230	Sense	TTC​GCA​GCC​TTA​GTC​CTC​AC
LOC108178230	Antisense	ATG​CTT​GAT​GTG​AGC​CTT​GGA
miR-29a-5p		ACT​GAT​TTC​TTT​TGG​TGT​TCA​GA
miR-30c-5p		TGT​AAA​CAT​CCT​ACA​CTC​TCA​GCT
miR-148a-5p		AAA​GTT​CTG​AGA​CAC​TCC​GAC​T
miR-375-5p		ACT​TGG​GCC​AAG​GGA​ATG​CAA​ACT
miR-450a-3p		ATT​GGG​AAC​ATT​TTG​CAT​GCA​T
miR-135a-3p		ATA​TAG​GGA​TTG​GAG​CCG​TGG​C
miR-450a-5p		TTT​TGC​GAT​GTG​TTC​CTA​ATA​T
miR-181d-3p		CCC​ACG​GGC​AGG​TGA​ATG​TCA​T

### Analysis of miRNA

TruSeq small RNA sample preparation kit (Illumina, San Diego, United States) was used to construct six small RNA libraries (CON-1, CON-2, CON-3, HFD-1, HFD-2, and HFD-3) according to the manufacturer’s instructions. Sequencing the library on the Illumina HiSeq 2500 platform (Illumina, San Diego, United States), SOAPnuke software (https://github.com/BGI-flexlab/SOAPnuke) was used to filter sequencing readings (Shao et al., 2021), and Bowtie2 was used to align 18 nt or larger small RNA readings with the rabbit reference genome (http://bowtie-bio.sourceforge.net/bowtie2/manual.shtml) and com-pared with Rfam database (http://rfam.xfam.org), GenBank database (https://www.ncbi.nlm.nih.gov/genbank/), and RepBase databases (https://www.girinst.org/repbase) removed known types of RNA sequences and repeats (Geisler & Renquist, 2017; Parry et al., 2020; Bai et al., 2021). Next, the software package miRDeep2 2.0.0.8 (https://github.com/rajewsky-lab/mirdeep2) was used to identify novel miRNAs from unmodified sequences (Vergani, 1969). Differential expression miRNAs were identified using the EdgeR data analysis package of R with a threshold of |log2 (fold change)| ≥ 1 and a *p* value < 0.05.

### Target gene prediction and enrichment analysis for miRNA

The miRNA targets prediction was performed by the software miRanda (http://www.microrna.org/microrna/home.do) and RNAhybrid (https://bibiserv.cebitec.uni-bielefeld.de/rnahybrid/), and the intersection of the predicted results was taken as the outcome (Jan and Marc, 2006). Next, the online software DAVID Bioinformatics Resources 6.7 (https://david.ncifcrf.gov/home.jsp) was used for GO and KEGG pathway enrichment analysis (Duvaud et al., 2021).

### Analysis of lncRNA

Sample RNA was prepared, the first cDNA strand was synthesized in M-MuLV reverse transcriptase system, the second cDNA strand was synthesized in dNTPs and DNA polymerase I, poly (A) tails were added and sequencing connectors were connected to generate 250–300 BP cDNA, and PCR amplificated to build the cDNA libraries using the NEBNext^®^ Ultra™ RNA Library Prep Kit for Illumina^®^ (New England Biolabs, Ipswich, MA, United States), following the manufacturer’s method. The cDNA library was sequenced using the Illumina HiSeq platform of Chengdu life baseline Technology co LTD. (Chengdu, China). The DEseq package from R was used for the differential expression analysis. The threshold was defined as |log2 (fold change)| ≥ 1 and *p* value ≤ 0.05.

### Target gene prediction and enrichment analysis for lncRNA

Combined with the correlation between lncRNAs and genes, the genes whose genome location overlapped with lncRNAs and the upstream and downstream 100 kb genes were selected as the candidate CIS target genes for lncRNAs regulation. Next, the potentially trans-regulated target genes of the DE lncRNAs were predicted based on the Pearson correlation coefficient. Then GO seq and R package were used for GO enrichment and KEGG pathway analysis of the candidate DE lncRNA target genes, respectively.

### Co-analysis of miRNA and lncRNA

MiRanda database (https://www.microRNA.org) was used to predict DE miRNAs related target genes. The database was one of the few software that could directly input sequences for prediction. It mainly emphasized the evolutionary conservation of the connecting sites between miRNAs and target genes, so it was widely used. The target genes were then overlapped with DE lncRNAs to screen the interaction pairs of DE miRNAs (upregulated) and DE lncRNAs (downregulated) or DE miRNAs (downregulated) and DE lncRNAs (upregulated). Finally, the molecular regulatory network was drawn by using the after-sales tool platform of Nuohe Zhiyuan company (https://magic.novogene.com/customer/main#/login).

### Statistical analysis

Statistical analysis was performed by using GraphPad Prism v5.0 software (San Diego, CA, United States). Differences between measurements from the two rabbit groups were assessed using Student’s *t* tests. Data were presented as means ± SEM. *p* < 0.05 was considered significant.

## Results

### Morphologic observation of rabbits’ liver

**FIGURE 1:**
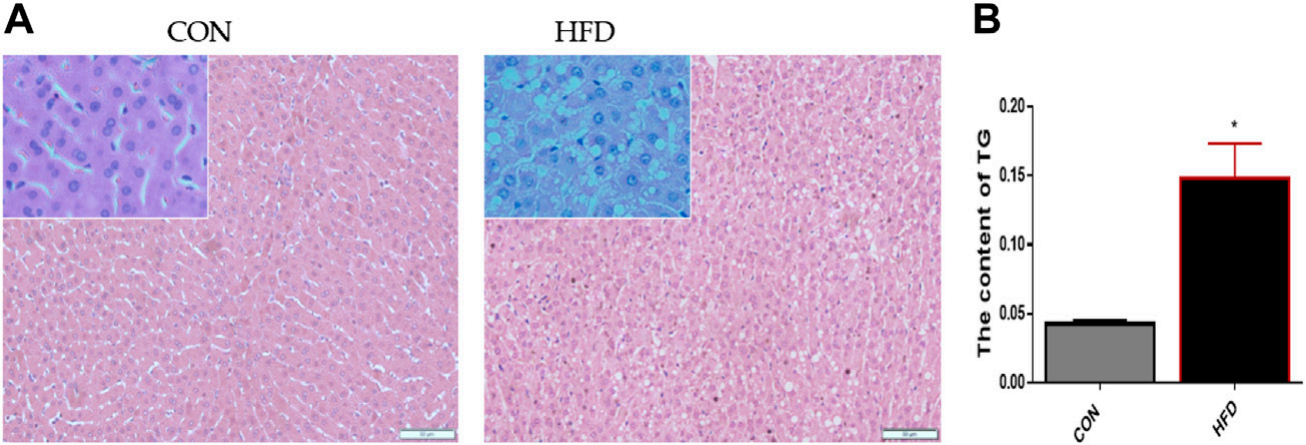
Morphologic observation of the CON and HFD rabbits’ liver **(A)** Paraffin section stained with HE showed the obvious difference between the liver tissues. **(B)** Triglyceride test results showed that there were significant differences. The data are presented as means ± SEM. *p < 0.05; **p < 0.01.

The results showed that the CON liver tissue structure was complete, the hepatocytes were arranged orderly, and there were no obvious histopathological differences. On the contrary, the HFD hepatocytes sections showed obvious steatosis, the number of lipid droplets around the nucleus increased, the hepatocyte arrangement was disordered (Figure 1A), and triglyceride content increased (Figure 1B).

### Quality assessment of miRNA sequencing

As shown in Table 2, a total of 62,449,703 clean reads were obtained from the six small RNA sequencing libraries when the raw reads were quality filtered. The CON and HFD groups had comparable levels of Q20 (percentage of reads with a Phred quality value > 20) with ranged from 99.59% to 99.85%. The GC content of libraries ranged from 44.98 % to 45.52% with an average content of 45.22%. Moreover, the mapped rate (the clean reads were mapped to the rabbit reference genome) had been further studied, and the mapped rate of all samples was higher than 93.4%. Therefore, all libraries were of high quality and could be used for further analysis.

**TABLE 2:** Statistics of miRNA data output quality of CON and HFD rabbits.

Sample	Clean reads	Q20 (%)	GC (%)	Mapped genome(%)
CON1	10,473,567	99.85	45.08	64.59
CON2	10,224,598	99.59	45.52	73.39
CON3	10,312,663	99.62	45.13	69.22
HFD1	10,424,133	99.84	44.98	64.49
HFD2	10,611,101	99.71	45.33	64.36
HFD3	10,403,641	99.68	45.29	66.38

### Identification of differentially expressed miRNA

**FIGURE 2:**
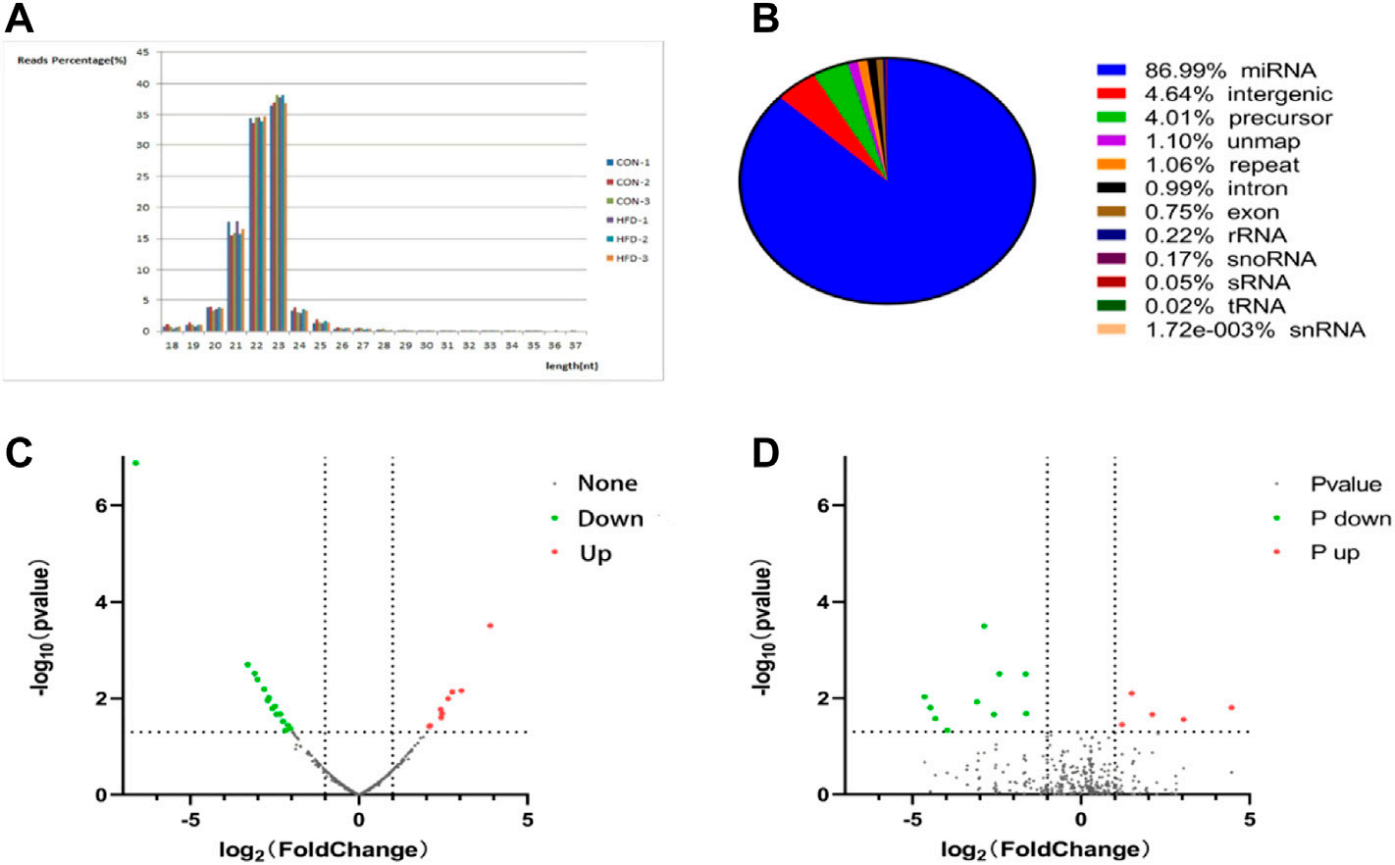
Identification of DE miRNA **(A)** Tags length distribution of liver samples. **(B)** Quantitative statistical results of the known DE miRNAs and the novel DE miRNAs. The volcano plot was constructed for the known **(C)** and the novel **(D)** DE miRNAs based on log2(fold change) and -log10(*p*-value).

**FIGURE 3:**
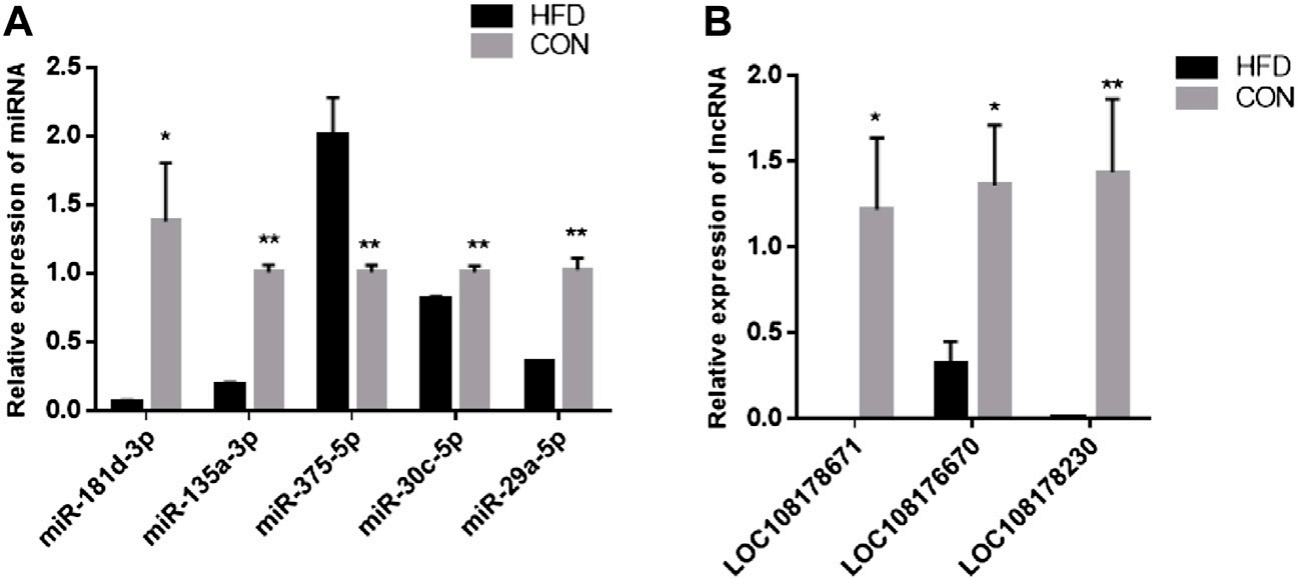
Validation of DE miRNAs **(A)** and DE lncRNAs **(B)**. The data are presented as means ± SEM. *p < 0.05; **p < 0.01.

The sequence length of miRNA was analysed, and the results showed that the average length of most reads was 23 nt in the six small RNA libraries, which was consistent with the length characteristics of animal miRNAs (Figure 2A). Reads >18 nt were compared to the Rfam, GenBank, and Repbase databases, and the results were listed in Figure 2B. The number of high-quality miRNA sequences obtained for each sample were 9,121,967 for CON-1, 8,590,722 for CON-2, 9,060,088 for CON-3, 9,439,855 for HFD-1, 9,129,172 for HFD-2, and 8,985,083 for HFD-3 in Supplementary Table S1. Next, the possible miRNA reads were compared to mature rabbit miRNAs in the miRbase database to identify samples known miRNAs (Supplementary Table S2), these known miRNAs could be directly used for subsequent analysis. Thirty six known DE miRNAs (16 upregulated and 20 downregulated) and 15 novel DE miRNAs (5 upregulated and 10 downregulated) were identified (Figure 2A). Values of log2(fold change) and −log10(*p*-value) were used to construct volcano figures for known (Figure 2C) and novel (Figure 2D) differentially expressed miRNA. Moreover, five DE miRNAs were randomly selected for qRT-PCR validation, and the results showed a similar trend to that of microRNA sequencing (Figure 3A).

### Enrichment analysis of differentially expressed miRNA target genes

**FIGURE 4:**
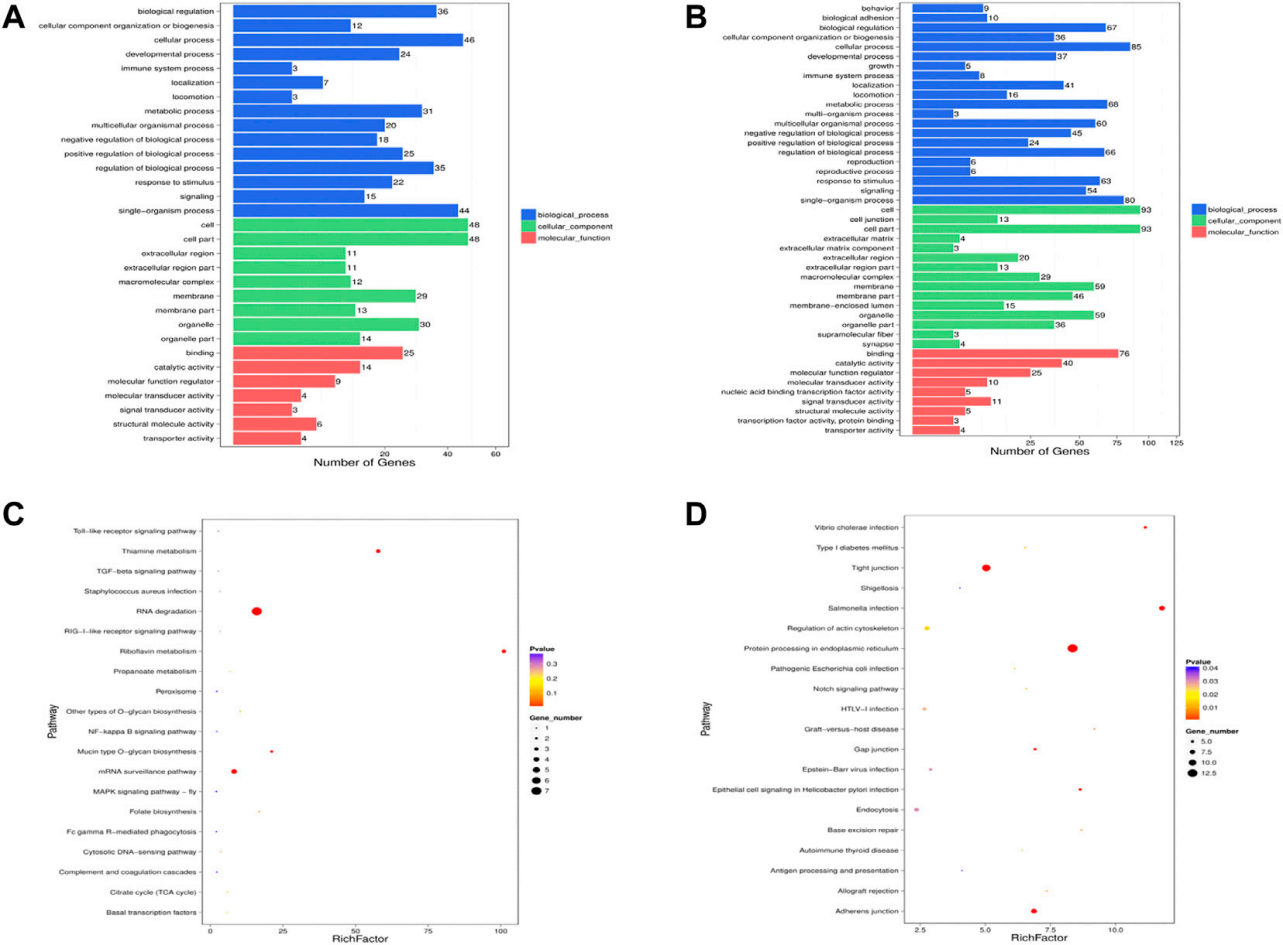
Enrichment analysis of the DE miRNAs target genes. GO analysis was performed for the known **(A)** DE miRNAs target genes and the novel **(B)** DE miRNAs target genes, and the terms with significant enrichment in BP, CC and MF categories were imaged. KEGG analysis was per-formed for known **(C)** DE miRNAs target genes and novel **(D)** DE miRNAs target genes, and only the top 20 significantly enriched pathways were listed.

Sixteen the known DE miRNAs target genes and 86 the novel DE miRNAs target genes were obtained. Go analysis results showed that there were 498 enriched GO items [341 biological processes (BP), 62 cell components (CC) and 96 molecular functions (MF)] in the known DE miRNAs target genes (Supplementary Table S3). The main biological processes involved in the known DE miRNAs target genes included lipid transporter activity, response to lipid, positive regulation of cell development, positive regulation of stem cell proliferation. Figure 4A showed the significantly enriched terms in the BP, CC, and MF categories. Among the novel DE miRNAs target genes, 789 BP, 490 CC and 179 MF were significantly enriched (Supplementary Table S4). Some GO terms related to development were significantly rich, included positive regulation of cell development and regulation of cell development (Figure 4B). Furthermore, the KEGG pathway analysis results showed that the known DE miRNAs target genes were enriched in 32 pathways, and RNA degradation, riboflavin metabolism, thiamine metabolism, mRNA surveillance pathway, mucin type O-glycan biosynthesis pathways were significantly enriched (Supplementary Table S5, Figure 4C). The novel DE miRNAs target genes were enriched in 77 pathways, including sphingolipid signalling pathway, Insulin resistance and glucagon signalling pathway were enriched and the top 20 significantly enriched pathways were presented in Figure 4D (Supplementary Table S6).

### Quality assessment of lncRNA sequencing

On average, each individual obtained 80,494,151 high-quality clean reads (range: 80,234,040–81,122,280), and the clean reads rate was 100%. Using HISAT to align the clean reads with the reference genome, the high localization efficiency was ≥91.26% (Table 3). These the known miRNAs could be directly used for subsequent analysis. Moreover, the window size was used to calculate the information distribution on each chromosome, and compared with the reference genome by reading. Finally, based on the existing rabbit reference gene annotation, the highly reliable lncRNAs was predicted (Supplementary Table S7).

**TABLE 3:** Statistics of lncRNA data output quality of CON and HFD rabbits.

Sample	Clean reads	Q20 (%)	GC (%)	Percentage of mapped reads (%)
CON1	80,806,448	97.49	51.48	91.74
CON2	80,283,856	97.74	52.20	92.22
CON3	80,234,040	97.00	51.64	91.26
HFD1	81,122,280	97.38	51.72	91.60
HFD2	80,244,782	97.50	51.50	91.70
HFD3	80,273,500	97.70	51.59	92.60

### Screening of DE lncRNAs and enrichment analysis of target genes

**FIGURE 5:**
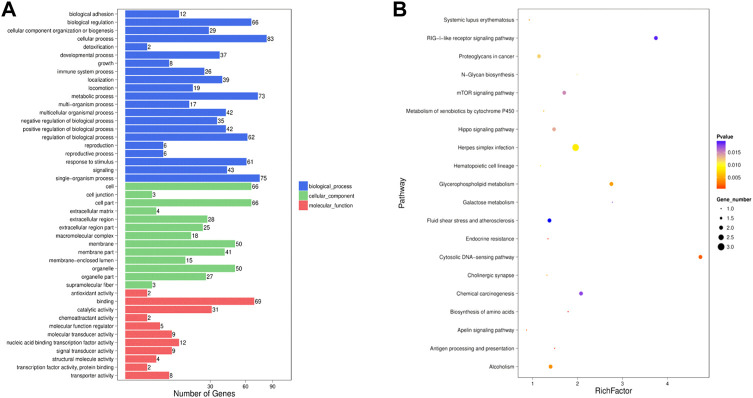
Screening of the DE lncRNAs and enrichment analysis of target genes **(A)** GO analysis of the DE lncRNAs were performed, imaging only those terms that were significantly enriched in the BP, CC, and MF categories. **(B)** KEGG analysis of the DE lncRNAs only listed the first 20 significant enrichment pathways.

The 24,615 DE lncRNAs were identified at *p* value ≤ 0.05. Of these, 10,851 (44%) were upregulated and 13,764 (56%) were downregulated in the HFD liver (Supplementary Table S8). Three DE lncRNAs were randomly selected for qRT-PCR validation, and the results showed a similar trend to that of lncRNA sequencing (Figure 3B). Go analysis results showed that 1,332 GO items were significantly enriched [783 biological processes (BP), 396 cellular components (CC) and 153 molecular functions (MF)] (Figure 5A). Furthermore, KEGG analysis results showed that some pathways related to lipid metabolism and immune diseases were enriched. Figure 5B showed 20 enriched pathways, some of which were related to adipocyte growth. 1,148 potential cis-acting lncRNAs target genes and 7,56,155 trans-regulation lncRNAs target genes were predicted. These data suggested that the most DE lncRNAs at different growth stages were related to lipid metabolism.

### Analysis of lncRNA-miRNA molecular regulatory network

**FIGURE 6:**
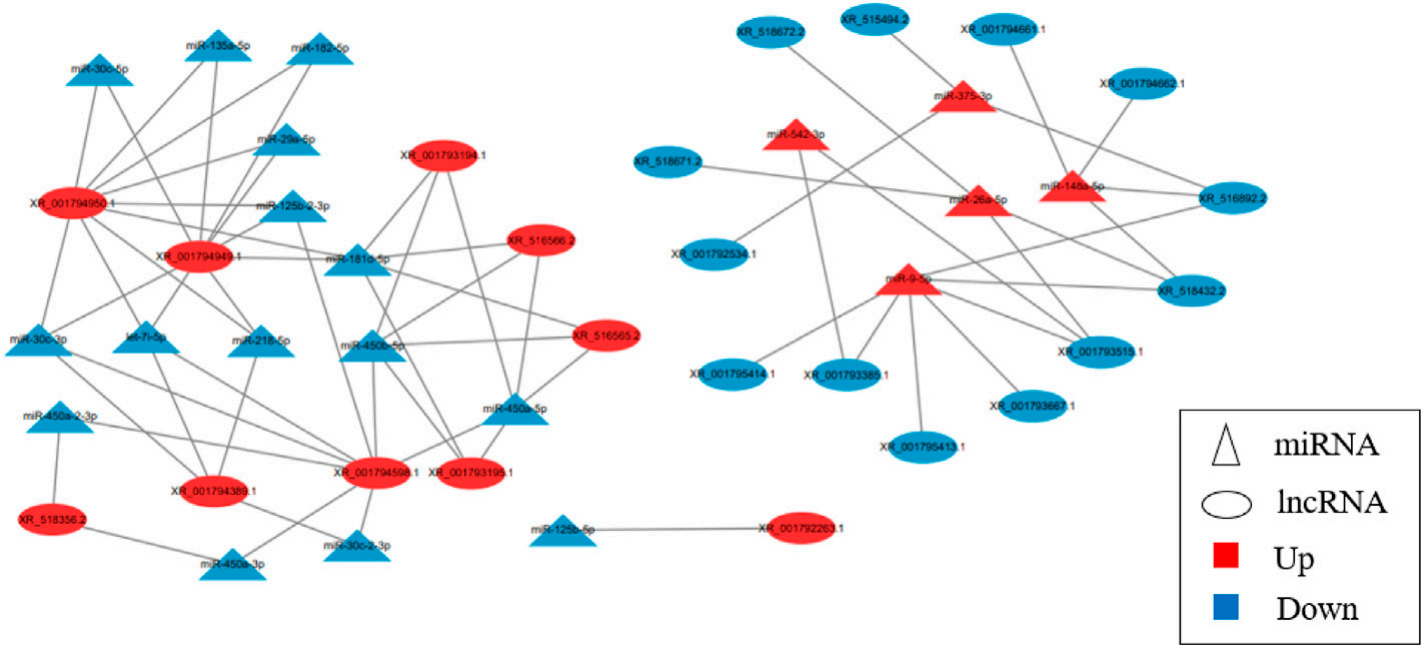
LncRNA-miRNA molecular regulatory network.

**FIGURE 7:**
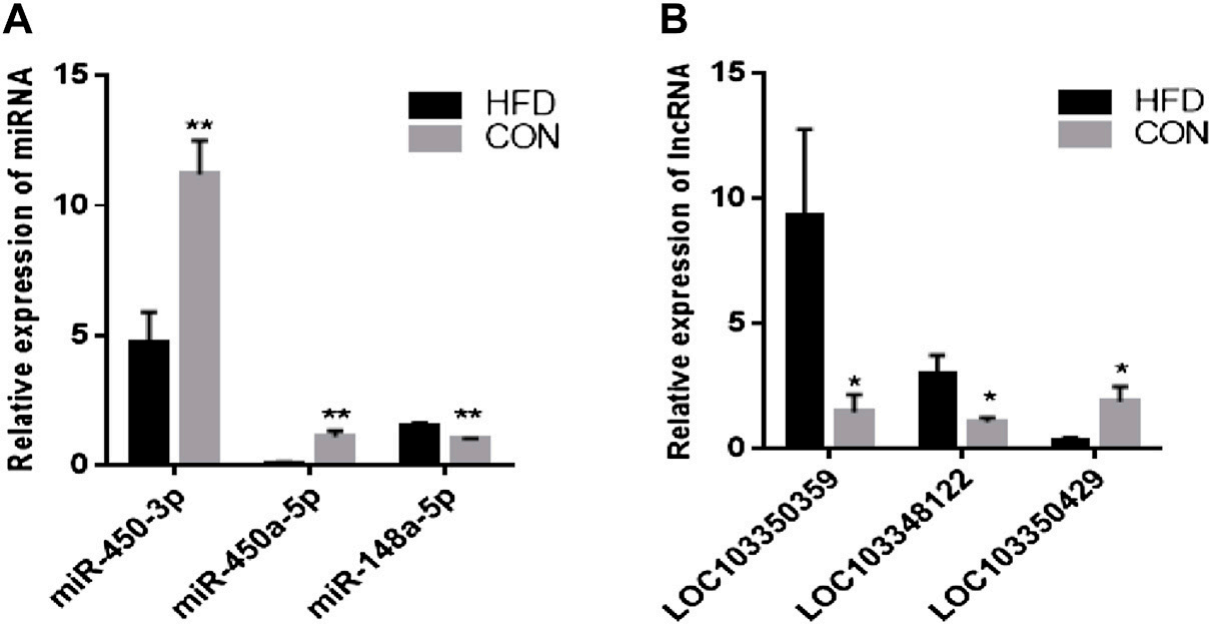
Validation of DE miRNAs and DE lncRNAs. The data are presented as means ± SEM. *p < 0.05; **p < 0.01.

The results showed that five up-regulated DE miRNAs could control 9 downregulated DE lncRNAs, 16 downregulated DE miRNAs could affect seven upregulated DE lncRNAs, and the lncRNA-miRNA molecular regulatory diagram with 43 nodes and 65 target relationships had been established (Figure 6). Among them, miR-9-5p combined six lncRNAs, while miR-125b-5p, miR-30c-5p, miR-450a-3p, miR-450a-2-3p, miR-135a-5p and miR-182-5p only linked one lncRNA respectively. Moreover, LOC103348122/miR-450a-5p, LOC103350359/miR-450a-3p and LOC103350429/miR-148a-5p were proposed the first time, then they were selected for qRT-PCR validation, the results showed a similar trend to that of miRNAs and lncRNAs sequencing (Figure 7). It was worth noting that miR-450a-5p and miR-148a-5p were related to lipid metabolism in the liver.

## Discussion

In higher vertebrates, the liver is the main metabolic organ and plays an important role in the process of lipid metabolism (Eduardo et al., 2014; Chadt & Al-Hasani, 2020). With the improvement of people’s living standards, such as high-fat diet, lipid accumulation in the liver exceeds the metabolic capacity of the liver itself, leading to metabolic diseases. Rabbit is an ideal model to study human diseases because it has similar lipid metabolic pathways (Bai et al., 2021; Shao et al., 2021). Here, our aim is to establish an obese rabbit model through a high-fat diet, identify the expression differences of lncRNAs and miRNAs in the process of liver steatosis through high-throughput sequencing, and try to understand the key lncRNA-miRNA molecular regulation mechanism of liver fat accumulation.

White cavities around the nuclei were found by HE staining, accompanied by an increase in liver TG levels (Supplementary Table S9). In fact, in the case of over nutrition and obesity, hepatic fatty acid metabolism changes (Parry et al., 2020), which usually leads to the accumulation of triglycerides in hepatocytes and a large amount of adipose deposition in cells to form lipid droplets; The cell volume increases with the increase of lipid, accompanied by the expansion of cell membrane, resulting in cell arrangement confusion (Vergani, 1969; Geisler & Renquist, 2017; Broadfield et al., 2021). This result is similar to that of Franco-Mahecha & Carrasco (2021).

According to miRbase standard, 51 DE miRNAs were identified. Among them, miR-107, miR-133, and miR-182-5p participate in the related processes of lipid metabolism. MiR-107 induced triglyceride storage defects by impairing glucose uptake and triglyceride synthesis in mature adipocytes (Ahonen et al., 2019; Foley & O'Neill, 2012; Zhang et al., 2018). MiR-182-5p improved HFD induced nonalcoholic steatohepatitis by inhibiting toll like receptors (Liang et al., 2019). Fathi et al. (2020) provided a comprehensive view of the network transcription factors in which miR-133 plays a central role, and supported the related role of myomiRs in regulating physiological hypertrophy of the heart.

Two hundred and thirty five target genes of 28 DE miRNAs were obtained. Among them, CNOT1, SUCLG1, and FGF6 were related to lipid metabolism. CNOT1 encodes the necessary scaffold subunit of ccr4-not dead keratinase complex in adipose tissue to affect the function of adipose tissue (Takahashi et al., 2019; Takahashi et al., 2020). FGF6 is a fat factor, which can regulate UCP1 and regulate systemic energy metabolism through a transcriptional network separated from brown fat (Shamsi et al., 2020; Bo et al., 2021). Midha et al. (2012) changed that in mouse liver proteins were identified using iTRAQ, offline 2DLC (SCX and RP) and MALDI-TOF/TOF Ms. It was found that SU-CLG1 can be associated with the physiological state of obese T2D (Meierhofer et al., 2015). Go analysis results showed that GO terms including lipid transporter activity, lipid response, positive regulation of cell development and positive regulation of stem cell proliferation were significantly rich, indicating that DE miRNAs played a role in regulating adipogenesis. KEGG pathway analysis showed that RNA degradation, riboflavin metabolism, thia-mine metabolism, mRNA monitoring pathway, mucin O-glycan biosynthesis pathway, insulin resistance and glucagon signaling were significantly enriched. Insulin and glucagon could lead to the imbalance between energy intake and energy consumption, resulting in excessive accumulation of triglycerides in adipose tissue (Magalhães et al., 2019). Therefore, the known DE miRNAs rich in these pathways suggested that these DE miRNAs might be important regulators in adipogenesis.

According to the standard of DEseq2, 24615 DE lncRNAs were screened. Xia et al. (Xia et al., 2021) found that lncRNA mainly performs its function by regulating the expression of coding genes. There are two main modes of regulation: cis target gene regulation and trans target gene regulation. It has been reported that lncRNA can regulate genes that overlap with or near it (Beylerli et al., 2020). The genes overlapped with lncRNA at the genomic position and 100 kb upstream and downstream are selected as candidate targets for lncRNA regulation, and 1,148 cis target genes are obtained. lncRNA could also remotely influence gene expression through trans action. The highly correlated lncRNA and mRNA were selected for sequence similarity analysis, and 2,151 trans acting target genes were obtained. Go analysis results revealed high concentration related to lipid metabolism. KEGG analysis results showed that insulin pathway entries were significantly enriched. It could cause excessive accumulation of lipid droplets and triglycerides in adipose tissue by regulating glucose metabolism (Aleixandre et al., 2018).

Noncoding RNA molecules, especially miRNAs and lncRNAs, are very common regulatory molecules. The molecular regulatory mechanism between them has been proved to play a variety of roles in many biological processes (Paraskevopoulou & Hatzigeorgiou, 2016; Yang et al., 2020; Dimitra et al., 2021). Some studies had found that the molecular regulation of miRNA and lncRNA changed during chicken obesity, and lncRNA-miRNA axis might be an important regulator of chicken abdominal fat expression (Li et al., 2015; Zhai et al., 2021). In this study, it was found that lncRNAs could target multiple miRNAs in the molecular regulatory network. The molecular regulation mechanism between five miRNAs and lncRNAs were found for the first time, it was worth noting that miR-450a-5p and miR-135a-3p were related to lipid metabolism in the liver.

The molecular mechanism of LOC103348122/miR-450a-5p and LOC103350429/miR-148a-5p involved in lipid metabolism is still largely unknown. Some studies found that miR-450a-5p could regulate fat formation by paracrine various factors (Wei et al., 2020; Chen et al., 2020). It could inhibit the expression of WISP2 by targeting its 3′-UTR, thereby promoting the occurrence of fat. MiR-450a-5p also could be used as a potential target to improve insulin resistance and treat patients with diabetes related diseases (Wei et al., 2020). In addition, the expression of miR-148a in nonalcoholic fatty liver disease decreased (Wang et al., 2018) (Ma et al., 2020) found that miR-148a-5p was downregulated during abdominal preadipocyte differentiation in chickens, which was consistent with the results of this study. In a word, lncRNA-miRNA might play an important role in the occurrence and development of lipid metabolism. However, its function needs further verification.

## Conclusion

24,615 DE lncRNAs and 52 DE miRNAs were identified and their target genes were predicted respectively, including target genes of 7,56,904 lncRNAs and 235 miRNAs. Moreover, GO and KEGG analysis results showed that enriched the pathways related to lipid metabolism, including mucin O-glycan biosynthesis pathway, insulin resistance and glucagon signalling pathway. Moreover, 65 targeting relationships were obtained, among three novel lncRNA-miRNA molecular regulatory networks were found for the first time. Therefore, further functional verification is required in the future.

## Data Availability

The datasets presented in this study can be found in online repositories. The names of the repository/repositories and accession number(s) can be found in the article/Supplementary Material.

## Supplementary material

SUPPLEMENTARY TABLE S1 sRNA annotation result statistics (xlsx format).

SUPPLEMENTARY TABLE S2 miRNA identification results (xlsx format).

SUPPLEMENTARY TABLE S3 GO analysis of the known DE miRNAs (xlsx format).

SUPPLEMENTARY TABLE S4 GO analysis of the novel DE miRNAs (xlsx format).

SUPPLEMENTARY TABLE S5 KEGG enrichment analysis of the known DE miRNAs (xlsx format).

SUPPLEMENTARY TABLE S6 KEGG enrichment analysis of the novel DE miRNAs (xlsx format).

SUPPLEMENTARYTABLE S7 LncRNAs identification statistics (xlsx format).

SUPPLEMENTARYTABLE S8 24615 differentially expressed lncRNAs. (xlsx format).

SUPPLEMENTARY TABLE S9 Raw data of triglyceride test. (xlsx format).
